# Microfluidically Frequency-Reconfigurable Quasi-Yagi Dipole Antenna

**DOI:** 10.3390/s18092935

**Published:** 2018-09-04

**Authors:** Syed Imran Hussain Shah, Sungjoon Lim

**Affiliations:** School of Electrical and Electronics Engineering, College of Engineering, Chung-Ang University, 221, Heukseok-Dong, Dongjak-Gu, Seoul 156-756, Korea; engr.shahsyedimran@gmail.com

**Keywords:** frequency reconfigurable antenna, pneumatic micropump, Yagi antenna

## Abstract

In this paper, a frequency reconfigurable quasi-Yagi dipole antenna is proposed by leveraging the properties of microfluidic technology. The proposed antenna comprises a metal-printed driven dipole element and three directors. To tune resonant frequencies, microfluidic channels are integrated into the driven element. To maintain a high gain for all the tuned frequencies, microfluidic channels are also integrated into the directors. Therefore, the length of the driven-element as well as directors can be controlled by injecting liquid metal in the microfluidic channels. The proposed antenna has the capability of tuning the frequency by varying the length of the metal-filled channels, while maintaining a high gain for all the tuned frequencies. The proposed antenna’s performance is experimentally demonstrated after fabrication. The injected amount of liquid metal into the microfluidic channels is controlled using programmable pneumatic micropumps. The prototype exhibits continuous tuning of the resonant frequencies from 1.8 GHz to 2.4 GHz; the measured peak gain of the proposed antenna is varied in the range of 8 dBi to 8.5 dBi. Therefore, continuous tuning with high gain is successfully demonstrated using liquid-metal-filled microfluidic channels.

## 1. Introduction

The Yagi-antenna has received particular attention due to its interesting features, like simple structure, low profile, ease of fabrication and installation [1], high radiation efficiency [2], and unidirectional radiation with high gain [3,4,5,6]. Reconfigurable antennas are important for the size reduction of multifunctional communication systems. Various tuning technologies can be used to realize frequency-reconfigurable antennas using PIN diodes [7], varactor diodes [8,9], ferroelectric varactors [10], and microelectromechanical systems (MEMS) switches [11,12]. Previously, a few frequency reconfigurable antennas have been reported by using ideal switches and varactor diodes. For instance, a frequency switchable Yagi antenna was presented by using ideal switches [13]. The operational frequency of the antenna was switched between 2.4 GHz and 5.78 GHz. A frequency-reconfigurable quasi-Yagi antenna was presented by employing varactor diodes in the driven elements as well as in the directors [14]. The resonant frequency of the antenna was tuned from 1.8 GHz to 2.4 GHz. The measured reported gain of the antenna was 5.6 dBi to 7.6 dBi. In [15], a frequency tunable Yagi antenna was presented aimed for wireless body area network applications. The frequency tuning feature was achieved by using PIN diodes in the driven element as well as in the parasitic elements. In [16], a frequency reconfigurable Yagi-Uda array was presented to serve as base station antennas. PIN diodes were installed in the driven elements and in the directors to tune the frequency and to achieve a high gain for each frequency. A frequency tunable Yagi antenna for low frequency applications was presented. Varactor diodes were used in the driven element and in the directors for continuous tuning of resonance frequency [17]. A frequency and pattern reconfigurable Yagi antenna was presented in [18]. Antenna consists of meandered line driven element and parasitic elements. However, tuning range of the antenna was only 200 MHz. In [19], a reconfigurable Yagi-Uda monopole antenna was presented. Antenna consists of vertical tubing to realize the monopole and parasitic elements. However, structure of the antenna was very unstable and radome was needed to use antenna for practical applications. Similarly in [20,21], some frequency reconfigurable Yagi-antennas were presented with only simulation results. Frequency tuning by PIN diodes and varactor diodes are very effective in providing high-speed tunability. However, they exhibit some drawbacks in terms of low frequency tunability range, reduced radiation efficiency, and lower power handling capability [22]. Nonlinear distortions are also caused by the semiconductor devices. Therefore, the fluidic and eutectic gallium–indium (EGaIn) can be utilized as alternatives for frequency-reconfigurable antennas to overcome the above mentioned limitations. Microfluidic-based reconfigurability is gaining attention owing to its interesting features of wide frequency tunability range, higher power handling capacity, and higher linearity [22]. Biasing networks that can interfere with radio-frequency (RF) are not required for frequency tuning by this strategy. Recently, frequency-reconfigurable antennas such as monopole [22], slot antenna [23], and patch antenna [24], have been presented taking advantage of EGaIn. In [25], a pattern reconfigurable loop antenna, and in [26,27], stretchable antennas have been reported by using EGaIn. In addition, frequency-reconfigurable filters [28], and frequency-selective surfaces [29], are also implemented using liquid metals. 

Herein, a novel microfluidically controlled frequency-reconfigurable antenna is proposed by leveraging the properties of microfluidic technology. The microfluidic channels are integrated with the driven element and the directors, whose effective length can be adjusted by injecting a liquid metal in the microfluidic channels. The proposed antenna is capable of tuning the frequency through varying length of the metal-filled channels, while maintaining the high gain for all the frequencies by controlling the length of directors. The structure has been fabricated, where liquid metal inside the microchannels is injected by pneumatic micropumps. The prototype exhibits continuous tuning of resonant frequencies from 1.8 to 2.4 GHz, thus encompassing multiple wireless communication systems. The measured gain of the antenna is also obtained between 8 dBi and 8.5 dBi. The antenna presents end-fire radiation pattern for all of the frequency bands.

## 2. Antenna Design

**Figure 1:**
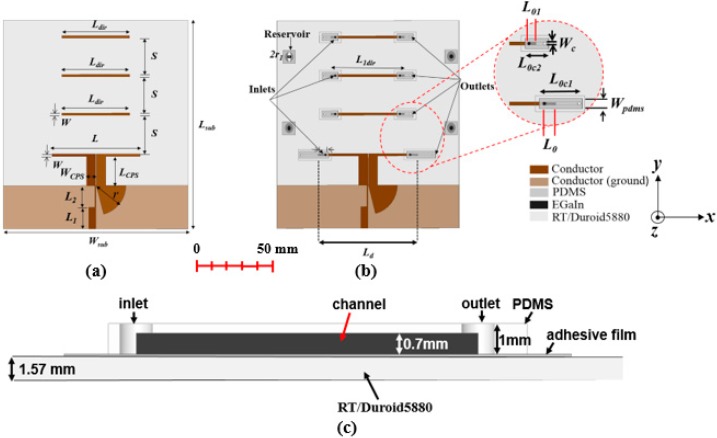
(**a**) Antenna geometry without microfluidic channels: L_sub_ = 120; W_sub_ = 110; L_1_ = 10; L_2_ = 10; r = 19; L_CPS_ = 19; W_CPS_ = 6; W = 1.5; L = 60; S = 25; and L_dir_ = 44. (**b**) Antenna geometry with microfluidic channels (partially filled) and reservoirs: L_d_ = 74; W_PDMS_ = 4.5; L_0c1_ = 14; L_0_ = 7; L_0c2_ = 5; L_01_ = 3; L_1dir_ = 50; W_c_ = 1.5 (units: mm), and (**c**) side view of the channel.

A microfluidically controlled frequency-reconfigurable quasi-Yagi antenna is designed as demonstrated in Figure 1. Rogers RT/Duroid 5880 substrate having a thickness of 1.57 mm and a relative permittivity (ε_r_) and loss tangent (tan δ) of 2.2 and 0.0009, respectively, is used for designing the proposed antenna. A driven element, three directors, and a microstrip to the co-planar-strip (CPS) broadband transition is designed on the top side of the antenna. A truncated ground plane is designed on the bottom side of the antenna.

The presented antenna is simulated and analyzed by using ANSYS High-Frequency Structure Simulator (HFSS). A microstrip to the CPS transition is used for impedance matching and field rotation. Furthermore, for matching impedance of microstrip line and coplanar strip line, impedance transformers are utilized. The antenna geometry is presented in Figure 1a.

Next, microfluidic channels are designed on the driven element and directors for injecting liquid metal to reconfigure the operating frequency and to maintain a high gain for each band. The detailed topology of the presented antenna with microfluidic channels is presented in Figure 1b. Microfluidic channels are fabricated using 1-mm-thick polydimethylsiloxane (PDMS), having ε_r_ and tan δ of 2.7 and 0.02, respectively [30]. For bonding PDMS on the driven element and directors, adhesive film having thickness and permittivity of 0.05 mm and 3, respectively, is used. The side view of the microfluidic channel is shown in Figure 1c. The depth of the microfluidic channel is 0.7 mm. The microfluidic channel is slightly overlapped on the driven element and directors; therefore, the liquid metal inside the microfluidic channels can be capacitively coupled to the driven element and directors through a thin bonding film and ensure the virtual RF short in the overlapped region.

A micropump is used for the injection of liquid metal in the microfluidic channels. Initially, some experiments were performed to select such a width of the microfluidic channel, so that the liquid metal can flow easily. For a wider channel width, the liquid metal can assumes a shape which cannot cover the entire width of the microfluidic channel. The optimized microfluidic channel’s width is 1.5 mm, which is the same width as that of the driven element and directors.

**Figure 2:**
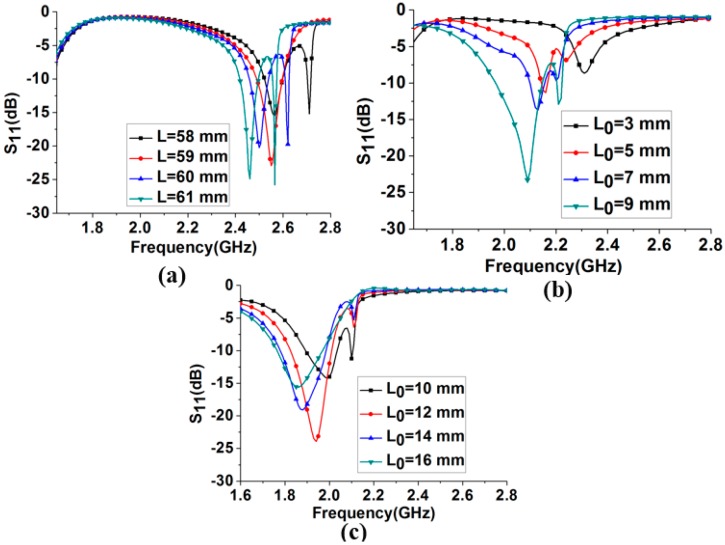
Simulated reflection coefficients by varying the driven element length at: (**a**) 2.4 GHz band; (**b**) 2.1 GHz band; and (**c**) 1.8 GHz band.

**Figure 3:**
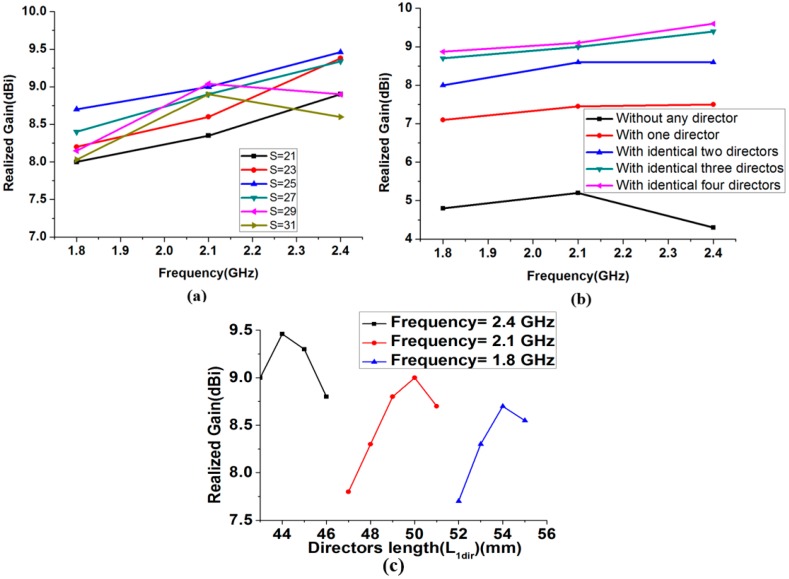
(**a**) Simulated realized peak gain for different S. (**b**) Simulated realized peak gain for all frequency bands for the different number of directors. (**c**) Simulated peak gain for different lengths of directors (L_1dir_) at 1.8 GHz, 2.1 GHz, and 2.4 GHz.

In the first state, with the unfilled microfluidic channels, the antenna is resonating at 2.48 GHz. The length of the driven element is determined from parametric study to obtain a resonant frequency of 2.48 GHz. In Figure 2a, the reflection coefficients are shown by changing L from 58 mm to 61 mm. The resonant frequencies for L = 58, 59, 60, and 61 mm corresponds to 2.57, 2.54, 2.48, and 2.46 GHz, respectively. Based on this study, the length of the driven element (L = 60 mm) is selected to obtain the desired operational frequency of 2.48 GHz. Next, the driven-element length is varied by the injecting liquid metal in the microfluidic channel of the driven element to obtain the operating frequencies of the other desired bands. The lengths of the adjustable directors are varied accordingly to obtain the maximum gain for each frequency band. In Figure 2b,c, the parametric study is performed for the length (L_0_) of the liquid metal in the microfluidic channel associated with the driven element to obtain the operating frequency of the other bands. By analyzing this study, L_0_ is selected as 7 mm and 14 mm to obtain the desired operating frequency of 2.1 GHz and 1.8 GHz, respectively. The length of the injected liquid metal in the driven element (L_0_) corresponding to each resonant frequency are listed in the Table 1. The distance between the reflector and driven element is optimized as L_CPS_ = 19 mm; this distance is selected after inspecting the directivity for all the desired bands. All the directors’ length and the spacing between driven element and directors are optimized with respect to the peak gain for all the desired bands. In Figure 3a, the simulated gain is plotted for various values of spacing (S) between the driven element and directors, for all operating frequencies. The realized gain at 2.4 GHz is varied from 8.6 to 9.46 dBi by varying S from 21 to 31 mm.

Therefore, the optimized value of S = 25 mm is selected for the maximum gain at all the desired operating frequencies. The gain of the proposed antenna versus the number of directors is plotted for all the frequency bands, as presented in Figure 3b. For instance, at 2.48 GHz, without any director, the antenna gain is 4.8 dBi.

**Figure 4:**
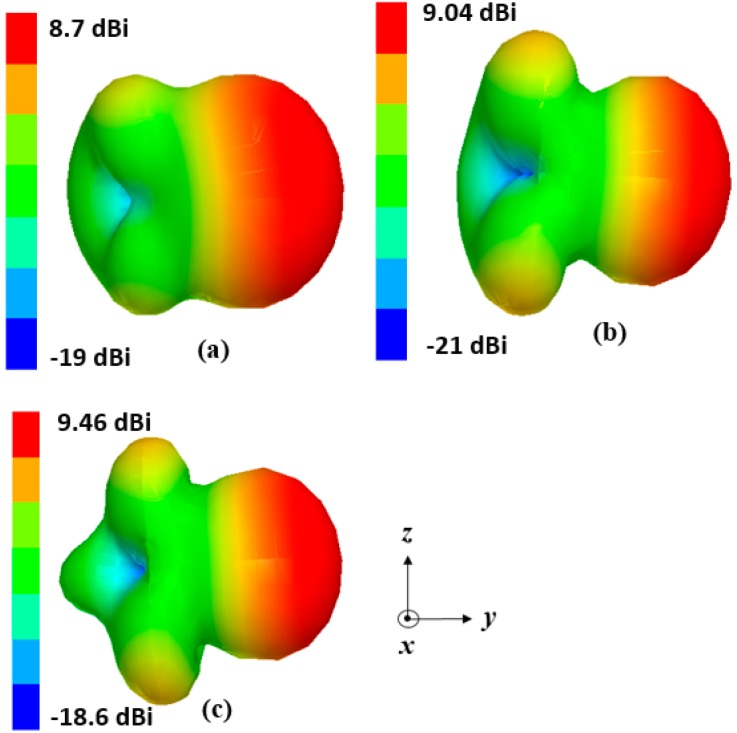
Simulated 3-D radiation pattern of final antenna design at: (**a**) 1.87 GHz; (**b**) 2.1 GHz; and (**c**) 2.48 GHz.

The antenna gain with one and two directors are 7.5 and 8.6 dBi, respectively. With the addition of three directors, the antenna gain increased up to 9.4 dBi. The antenna gain is significantly increased with three directors. With the addition of a fourth director, the increase in the antenna gain was not substantial for all the frequency bands. Therefore, only three directors are selected. The dimensions of three directors are identical. In the microfluidic channel of the directors, liquid metal is injected and their length (L_1dir_) is optimized to obtain the maximum peak gain for each resonant frequency. The length of the injected liquid metal in the director (L_01_ and L_1dir_) corresponding to highest peak gain are listed in the Table 1. Figure 3c shows the simulated peak gain for different lengths of directors (L_1dir_) at 1.8 GHz, 2.1 GHz, and 2.4 GHz. The optimized length of L_1dir_ is selected as 44 mm, 50 mm, and 54 mm for the 2.48 GHz, 2.1 GHz, and 1.8 GHz, respectively. After optimizing all the parameters, including the number and length of directors, spacing between reflector and driven element, spacing of driven-element and directors, and spacing of directors, the simulated version of antenna’s 3-D radiation patterns are plotted at 1.8, 2.1, and 2.4 GHz, as shown in Figure 4. The simulated peak gain of the antenna is 8.7, 9, and 9.4 dBi at 1.87, 2.1, and 2.48 GHz, respectively.

## 3. Experimental Verification

**Figure 5:**
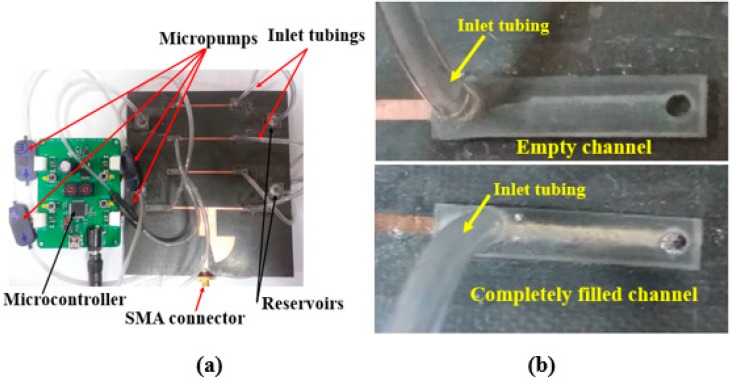
Pictures of (**a**) the fabricated prototype of the frequency-reconfigurable antenna and (**b**) magnification of empty, and completely filled channels.

**Figure 6:**
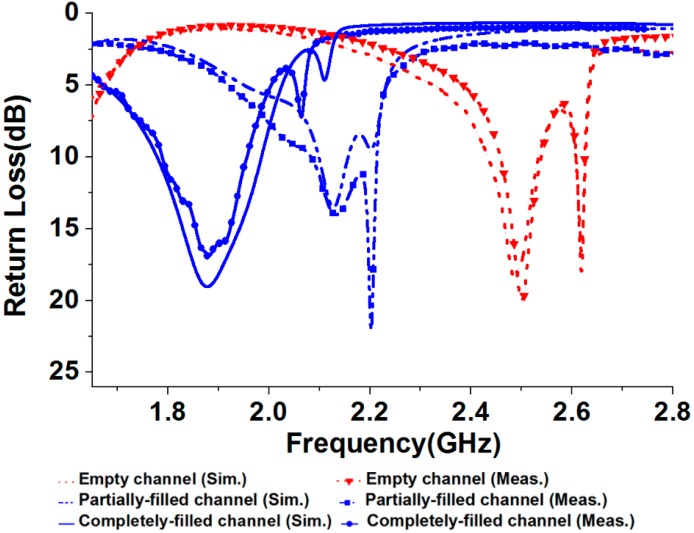
Simulated and measured return losses of the presented quasi-Yagi antenna.

**Figure 7:**
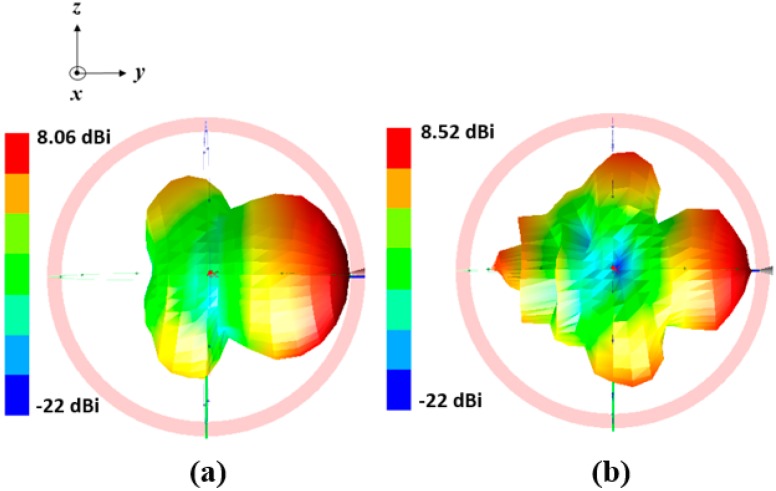
Measured 3-D radiation pattern of the antenna at (**a**) 1.87 GHz and (**b**) at 2.48 GHz.

The final antenna prototype is presented in Figure 5a. The magnified version of the microfluidic channels with empty, and completely filled states are presented in Figure 5b. The liquid metal from the reservoirs is injected in the microfluidic channels through tubes. The mp-6 micropumps provided from Bartels Mikrotechnik GmbH were utilized for the injection of liquid metal from the reservoirs in the microfluidic channels. The weight and dimensions of the micropump are 2 g and 30 mm × 15 mm × 3.8 mm, respectively and the power consumption is approximately 50 mW. To increase the micropump speed and to make it bidirectional, two micropumps can be connected in series. The control unit (mp6-OEM controller) consists of a microcontroller and the micropump’s driving circuit, which can be used to control four mp6 micropumps simultaneously with a single setting. The pump voltage and pump frequency can be directly changed with the rotary control elements of the board. The liquid metal has a propensity to form solid oxide skins on its surface when it is exposed to air/oxygen. These oxide layers adhere to the surface of the microfluidic channels and renders reconfiguration challenging. However, with proper surface treatments, these problems can be easily overcome [22,23]. Various solutions can be used for this purpose. For instance, hydrochloric acid (HCl) can be used to avoid the oxide from forming. EGaIn channels can be pre-treated with Nafion, then soaked in the HCl solution and rinsed. Nafion absorbs HCl and gradually releases it in vapor form, which avoids the formation of oxides. In order to avoid radiation perturbation from the liquid metal in the tube, the tube size is designed shorter than λ/10. The simulated and measured return losses of the proposed frequency reconfigurable antenna are shown in Figure 6. For the empty channels state, the resonant frequency of the antenna is 2.48 GHz. For the partially and completely injected microfluidic channels, the operating frequency of the antenna is 2.1 GHz and 1.87 GHz respectively. First, L_0_ is determined for the desired resonant frequency. Next, L_01_ is determined to achieve highest peak gain corresponding to each resonant frequency. Therefore, the operating frequency of the antenna can be continuously tuned from 1.8 GHz to 2.5 GHz and its relative tuning bandwidth is 35% while keeping highest peak gain. The simulated and measured S_11_ are in good agreement with each other. The shift in the resonance frequency of the second peak in the completely filled channels is due to the fabrication tolerance. The antenna is matched for all the desired bands without requiring any external matching circuitry, which simplifies the antenna design. The presented antenna can be suitable for various wireless communication systems, including personal communication systems (PCS), global system for mobile communications (GSM), and IEEE 802.11b wireless local area network (WLAN). The 3-D radiation pattern was measured in a shielded radio frequency anechoic chamber [31] at the lower and upper frequency bands and presented in Figure 7a,b.

The measured gain of the presented antenna is 8.5 dBi and 8 dBi at 1.87 GHz and 2.48 GHz, respectively. These measured gain values are 0.2 dBi and 1.4 dBi lower than the simulated gain of Figure 4 at the respective frequencies. The simulation and measurement results are slightly different which is possibly because of inaccuracy in the PDMS loss tangent. In the lower and upper band, measured efficiency of the presented antenna is 85% and 79%, respectively. When we measure the radiation pattern, we removed the microcontroller while keeping the tubes. The reconfiguration time for the entire tuning range was measured as 1.5 s when each channel is integrated with a micropump. At all the frequencies, the antenna has the end-fire radiation pattern. Furthermore, the null of the pattern disappears which is due to spurious radiation of the CPS lines, which is added to the dipole radiations. We experimentally observed that liquid-metal movement in the channels can be instantaneously started and stopped by switching the micropumps on and off, without any overshoot.

## 4. Conclusions

A microfluidically frequency-reconfigurable quasi-Yagi dipole antenna is presented by employing microfluidic technology. To tune the resonant frequencies, the microfluidic channels are integrated into the driven element and three directors. The length of driven element and directors can be controlled by the injection of liquid metal in microfluidic channels. The proposed antenna is capable of tuning the frequency by varying the length of the metal-filled channels of the driven element and directors while maintaining the high gain for all the tuned frequencies. The proposed antenna’s performance is experimentally demonstrated after fabrication. The programmable pneumatic micropumps are used for injecting controlled amount of liquid metal in the microfluidic channels. The prototype exhibits the continuous tuning of resonant frequencies from 1.8 GHz to 2.4 GHz and the measured peak gain varied in the range of 8 dBi to 8.5 dBi. Therefore, continuous tuning with high gain is successfully demonstrated using liquid-metal-filled microfluidic channels. Channel length can be further increased in order to increase antenna’s tuning range. As a future work, the number of micropumps will be reduced by connecting all the fluidic channels of the directors and using dielectric solutions in the channels to prevent the connectivity of the different directors.

## Figures and Tables

**Table 1:** Dimensions of channels and liquid metal corresponding to each frequency.

L_0c_	L_0c1_	L_0_	L_01_	L_d_ = L + 2 × L_0_	L_1dir_ = L_dir_ + 2 × L_01_	Frequency
14	5	14	5	88	54	1.8
14	5	7	3	74	50	2.1
14	5	0	0	60	44	2.48

L_0c_: channel length of driven element; L_0c1_: channel length of directors; L_0_: liquid metal length in driven element; L_01_: Liquid metal length in directors; L_d_: effective driven-element length; L_1dir:_ effective length of the three directors, (all the lengths are in mm and frequency is in GHz).
